# Investigation of VIM-1-producing *Enterobacter* spp. across Switzerland: clonal dissemination and plasmid transmission

**DOI:** 10.1128/aac.01827-25

**Published:** 2026-05-05

**Authors:** Helena M. B. Seth-Smith, Mariella Greutmann, Jacqueline Findlay, Christiane Beckmann, Livia Berlinger, Silvio D. Brugger, Riccarda Capaul, Gilbert Greub, Eva Gruner, Peter M. Keller, Oliver Nolte, Tim Roloff, Sarah Tschudin-Sutter, Walter Zingg, Patrice Nordmann, Laurent Poirel, Adrian Egli, on behalf of the NARA Network

**Affiliations:** 1 Institute of Medical Microbiology, University of Zurich 27217, Zurich, Switzerland; 2 Swiss Institute of Bioinformatics 30489, Lausanne, Switzerland; 3 Swiss National Reference Center for Emerging Antibiotic Resistance (NARA) Network, University of Fribourg 27211, Fribourg, Switzerland; 4 Medical and Molecular Microbiology Unit, Faculty of Science and Medicine, University of Fribourg 27211, Fribourg, Switzerland; 5 Viollier, Infection Diagnostics, Allschwil, Switzerland; 6 Medical Microbiology, Laboratory Medicine, Cantonal Hospital Lucerne 30748, Lucerne, Switzerland; 7 Department of Infectious Diseases and Hospital Epidemiology, University Hospital Zurich, University of Zurich 27217, Zurich, Switzerland; 8 Central Laboratory, Cantonal Hospital Graubünden, Chur, Switzerland; 9 Institute of Microbiology, University of Lausanne & University Hospital Center 27213, Lausanne, Switzerland; 10 Medica Medizinische Laboratorien AG 206150 , Zurich, Switzerland; 11 Laboratory Medicine, Clinical Bacteriology/Mycology, University Hospital Basel 30262, Basel, Switzerland; 12 Division of Infectious Diseases, University Hospital Basel and University of Basel 30262, Basel, Switzerland; Universita degli Studi di Roma La Sapienza, Rome, Italy

**Keywords:** plasmid, outbreak, VIM-1, hospital epidemiology, public health, whole genome sequencing

## Abstract

Carbapenemase-producing Enterobacterales (CPE) are a major public health concern. Within the *Enterobacter cloacae* complex (ECC), the *bla*_VIM-1_ carbapenemase gene is frequently plasmid-borne, enabling inter-clonal and inter-species spread that complicates the detection and control of carbapenemase dissemination. We investigated an increase in VIM-1-producing *Enterobacter* spp. reported to the Swiss National Reference Center for Emerging Antibiotic Resistance (NARA) between 2022 and 2024 using high-resolution genomic methods. Between January 2022 and October 2024, *bla*_VIM-1_-positive *Enterobacter* spp. isolates from 39 patients, plus additional contemporary *bla*_VIM-1_-positive Enterobacterales, were analyzed. Whole-genome sequencing (Illumina) was performed for all isolates, with long-read sequencing (Oxford Nanopore Technologies) for 12 selected isolates. Species identification, genomic relatedness by MLST and cgMLST, and fine-scale plasmid characterization and comparison were performed. Most isolates were *Enterobacter hormaechei* (*n* = 37), alongside *Enterobacter kobei* (*n* = 1) and *Enterobacter ludwigii* (*n* = 1), distributed across 13 sequence types, excluding purely clonal dissemination. Hybrid assemblies showed *bla*_VIM-1_ located on several plasmid types. IncHI2 plasmids of 249–343 kb carrying additional antimicrobial resistance promoting genes including *mcr-9* predominated, spanning multiple ECC lineages and also present in two other species. Conjugation of these was confirmed experimentally, and within-host plasmid variation was observed. No further cases occurred after October 2024. We describe a multiclonal spread of *bla*_VIM-1_ Enterobacterales in Switzerland, mediated by IncHI2 plasmids. These findings highlight the need for plasmid-focused genomic surveillance to complement clonal typing in CPE outbreak investigations.

## INTRODUCTION

*Enterobacter* are generally opportunistic Gram-negative pathogens frequently implicated in hospital-acquired infections (1). The *Enterobacter cloacae* complex (ECC) encompasses multiple closely related species, including *E. cloacae*, *E. hormaechei*, and *E. asburiae*, that are often difficult to distinguish with classical diagnostic methods—biochemical or matrix-assisted laser desorption ionization–time of flight mass spectrometry (MALDI-TOF MS)—but are collectively significant in clinical settings (1). *Enterobacter* spp. rank among the top opportunistic pathogens, causing an estimated 7% of nosocomial infections in intensive care units in the United States (2). These bacteria have large genomes (~5 Mb) and exhibit high genetic and metabolic diversity, which can complicate species-level identification in clinical laboratories (3).

A major concern is the acquired resistance of *Enterobacter* spp. to carbapenems, which are often last-resort antibiotics for multidrug-resistant infections. In particular, carbapenem-hydrolyzing enzymes such as Verona Integron-encoded metallo-β-lactamase (VIM) can inactivate virtually all β-lactam antibiotics, including carbapenems, with the exception of aztreonam (4). The *bla*_VIM-1_ gene, first identified in the late 1990s, has disseminated widely via mobile genetic elements (5). It is usually embedded in class 1 integrons on plasmids, facilitating horizontal transfer (6, 7). Over the past two decades, *bla*_VIM-1_ has become the most prevalent VIM variant globally, accounting for roughly 75% of reported VIM-producing Enterobacterales cases worldwide (8). Its dissemination has been particularly pronounced in Europe, where VIM-producing Enterobacterales have caused numerous nosocomial outbreaks and are now considered endemic in some healthcare settings, as supported by large-scale genomic surveillance studies from France and Poland and broader European epidemiological analyses (8–12).

Transmission events involving carbapenemase-carrying isolates pose a serious infection control challenge, especially when resistance genes spread via plasmids. Unlike clonal outbreaks, in which a single bacterial strain transmits, plasmid-mediated dissemination can involve genetically diverse bacterial species of the same or even different genera sharing a resistance-carrying plasmid.

Multiple studies have documented plasmid-mediated dissemination of *bla*_VIM-1_ across genetically diverse Enterobacterales, highlighting the limitations of clonal outbreak definitions. In hospital settings, multiclonal and multispecies dissemination events have been reported in Europe, including IncHI2-mediated spread of *bla*_VIM-1_ across diverse *E. cloacae* complex lineages in Spain and multispecies dissemination driven by broad-host-range IncA plasmids in Italy (13, 14). Large-scale surveillance studies further support this pattern, with investigations from France and Poland demonstrating widespread circulation of carbapenemase-producing Enterobacterales across multiple species and sequence types, consistent with plasmid-mediated transmission rather than single-clone expansion (10, 11). Beyond hospital settings, *bla*_VIM-1_-carrying *Enterobacter* spp. have also been detected in environmental reservoirs such as wastewater, highlighting the potential for extra-clinical persistence and circulation of these resistance determinants, with possible implications for reintroduction into clinical settings (15). Outside Europe, related *bla*_VIM-1_- and *mcr-9*-carrying IncHI2 plasmids have additionally been identified in the food chain, including in *E. hormaechei* isolated from raw meat in Egypt, demonstrating the global distribution of such resistance plasmids (16).

These examples highlight the plasmid-mediated dimension of carbapenemase dissemination. When *bla*_VIM-1_ resides on broad-host-range plasmids (such as IncHI2, IncA), dissemination events may be difficult to detect, as they can manifest as resistant isolates belonging to disparate species or sequence types that nevertheless share a common resistance element (13, 14). Such scenarios can easily be missed if surveillance focuses only on clonal relatedness. Comprehensive molecular surveillance is therefore critical to detect these insidious, plasmid-driven transmission events before they become unmanageable.

An increase in the number of *bla*_VIM-1_-positive *Enterobacter* spp. isolates submitted to the Swiss National Reference Center for Emerging Antibiotic Resistance was observed from 2022 to 2024, raising suspicion of a potential outbreak in the country. In this study, we aimed to explore the epidemiology using molecular surveillance tools, including short- and long-read sequencing to determine the connections of a potential plasmid-mediated dissemination of *bla*_VIM-1_.

## MATERIALS AND METHODS

### Sample collection

*Enterobacter* spp. isolates carrying *bla*_VIM-1_, mainly from urine samples and rectal swabs, from 39 patients were submitted from several Swiss hospitals to NARA between January 2022 and October 2024. Duplicate isolates were obtained from 12 patients. Isolates carrying *bla*_VIM-1_ belonging to different species received by NARA during this period (*n* = 13) were also analyzed. From October 2024 to June 2025, no new cases were documented.

### Biochemical/immunochromatographic analysis

Production of carbapenemases was assessed using the Rapidec Carba NP test (17). Positive isolates were tested using the NG-Test Carba 5 (NG Biotech, Guipry, France), assessing the production of either NDM-, VIM-, IMP-, OXA-48-, or KPC-like enzymes.

### Conjugation experiments

Conjugation assays were performed using sodium azide-resistant *E. coli* J53 as the recipient strain. Three donor strains were used: N1137 (OXA plasmid control), NARACHVIM20, and NARACHVIM36. Briefly, donor and recipient strains were grown overnight in Luria Bertani (LB) broth and then mixed in a 1:3 ratio (donor:recipient) before spotting onto a 0.22 µm filter placed on LB agar. Plates were incubated at 25°C for 6 h. Growth was collected and resuspended in 0.85% NaCl and inoculated onto agar plates containing either 100 mg/L sodium azide and 8 mg/L ceftazidime, or ceftazidime only. Following 24 h of incubation at 37°C, successful transconjugants were confirmed by PCR amplification of the *bla*_VIM-1_ gene. Conjugation frequencies were calculated by dividing the number of transconjugants by the number of donors. PCR confirmation of *bla*_VIM-1_ presence was performed using gene-specific primers VIM-F: 5′-TTATGCCGCACCCACGCCTA-3′ and VIM-R 5′-CTGCTACTCGGCGACTGAGC-3′.

### Short read sequencing

DNA was extracted using the Maxwell RSC Blood DNA Kit (Promega). Short-read sequencing was performed on all isolates using Qiaseq FX library preparation (Qiagen) and PE150 sequencing on an Illumina NextSeq1000 platform to achieve a mean read depth in excess of 30×. Data were analyzed using the IMMense pipeline (18) (gitlab.uzh.ch/appliedmicrobiologyresearch/immense).

### Long-read sequencing

Oxford Nanopore Technology (ONT) sequencing was further performed on 12 selected isolates. To prepare the long-read libraries, the SQK-RBK114.24 barcoding kit was used. Pooled libraries were run on a R10.4.1 flow cell (FLO-MIN114, Oxford Nanopore Technologies) and sequenced with a GridION over 72 h; basecalling and de-multiplexing were implemented in MinKnow software version 24.06.15. The quality of the reads was evaluated using Nanoplot v1.43.0 (19) and is summarized in Table S1. Adapter trimming was performed using Porechop v0.2.4 (https://github.com/rrwick/Porechop). Read counts ranged from 12,561 to 133,534, with total yields between 99.2 and 590.2 Mb. Read N50 values ranged from 5.1 to 14.3 kb, and mean read quality scores from Q16.8 to Q17.5. Between 89.5 and 91.5% of reads were above Q15, and 27.6 to 44.3% were above Q20.

### Hybrid assemblies

Hybrid assembly of the Illumina and ONT reads was performed using both Unicycler v0.5.0 (20) and, in parallel, Flye v2.9.4 (21), Medaka v2.0.0 (https://github.com/nanoporetech/medaka), and Polypolish v0.6.0 (22). Comparing the completeness and size of assembled plasmids along with coverage statistics, we selected the Flye–Medaka–Polypolish assemblies for downstream analyses, with the exception of sample NARACHVIM07, for which a complete plasmid assembly could only be obtained using Unicycler hybrid assembly.

### Genome and plasmid typing

Species determination was performed using TYGS (23) (tygs.dsmz.de). MLST and core genome MLST (cgMLST) were performed using Ridom Seqsphere+ v10.0.1 with an *ad hoc Enterobacter cloacae* complex core genome schema defined in Ridom Seqsphere+ v4.1.6 using 43 reference genomes and based on 638 loci with a 10-allele cluster cutoff. Single nucleotide polymorphism (SNP) phylogenies were generated in CLC Genomics Workbench v25.0 (Qiagen) using parameters that differed from the defaults as follows: variant calling with 10× minimum coverage, 10 minimum count and 70% minimum frequency, and neighbor-joining SNP tree used 10× minimum coverage, 10% minimum coverage, 0 prune distance, and inclusion of multi-nucleotide variants (MNVs). Plasmid coverage data were also obtained from this mapping procedure.

### Plasmid classification

Incompatibility groups were classified with PlasmidFinder (24). We analyzed the plasmid hybrid assemblies with pling v2.0.1 (25), which compares plasmid structures based on gene or block order. First, pling represents each plasmid as a series of signed blocks derived from sequence alignments. It then calculates containment values (how much of one plasmid is contained within another, with lower values representing higher containment), and we retained only pairs with containment ≤0.5 for further comparison. For these plasmid pairs, pling estimates the minimum number of rearrangements or insertion/deletion (indel) events using the Double Cut and Join with Indels (DCJ-Indel) model required to transform one plasmid structure into the other. Finally, we constructed a plasmid network in which edges were annotated with both the containment and the DCJ-Indel distance, providing a combined measure of structural similarity.

### Antimicrobial resistance gene identification

Genes involved in antimicrobial resistance were identified using AMRFinderPlus v3.11.26 (26, 27) with database version 2023-11-15.1 within Ridom SeqSphere+.

## RESULTS

### Surveillance signal

An increase in *bla*_VIM-1_-carrying *Enterobacter* spp. isolates (Table S2) submitted to NARA was observed during 2022–2024, raising the possibility of a clonal dissemination. Those isolates exhibited resistance to almost all beta-lactams, including broad-spectrum cephalosporins, aztreonam, and carbapenems, and were resistant to aminoglycosides and fluoroquinolones.

### Genomic typing

**Fig 1:**
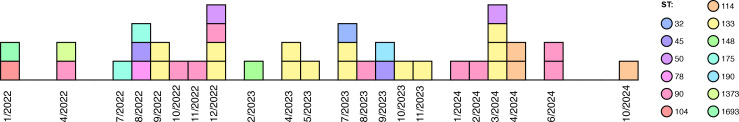
Epidemiological curve showing isolation dates of *n* = 39 *Enterobacter* spp., colored by ST. Only one isolate per patient is shown. In the few cases where the isolation date was not available, dates of reception at NARA were used as a substitute.

Genome assemblies were used to identify the bacteria as *E. hormaechei* (*n* = 37), *Enterobacter kobei* (*n* = 1), and *Enterobacter ludwigii* (*n* = 1). MLST showed 13 different sequence types within the data set, with the most common being *E. hormaechei* ST133 (*n* = 14), ST90 (*n* = 9), and ST114 (*n* = 3) (Fig. 1). These STs do not show clear temporal clustering patterns or geographical patterns within Switzerland (data not shown).

**Fig 2:**
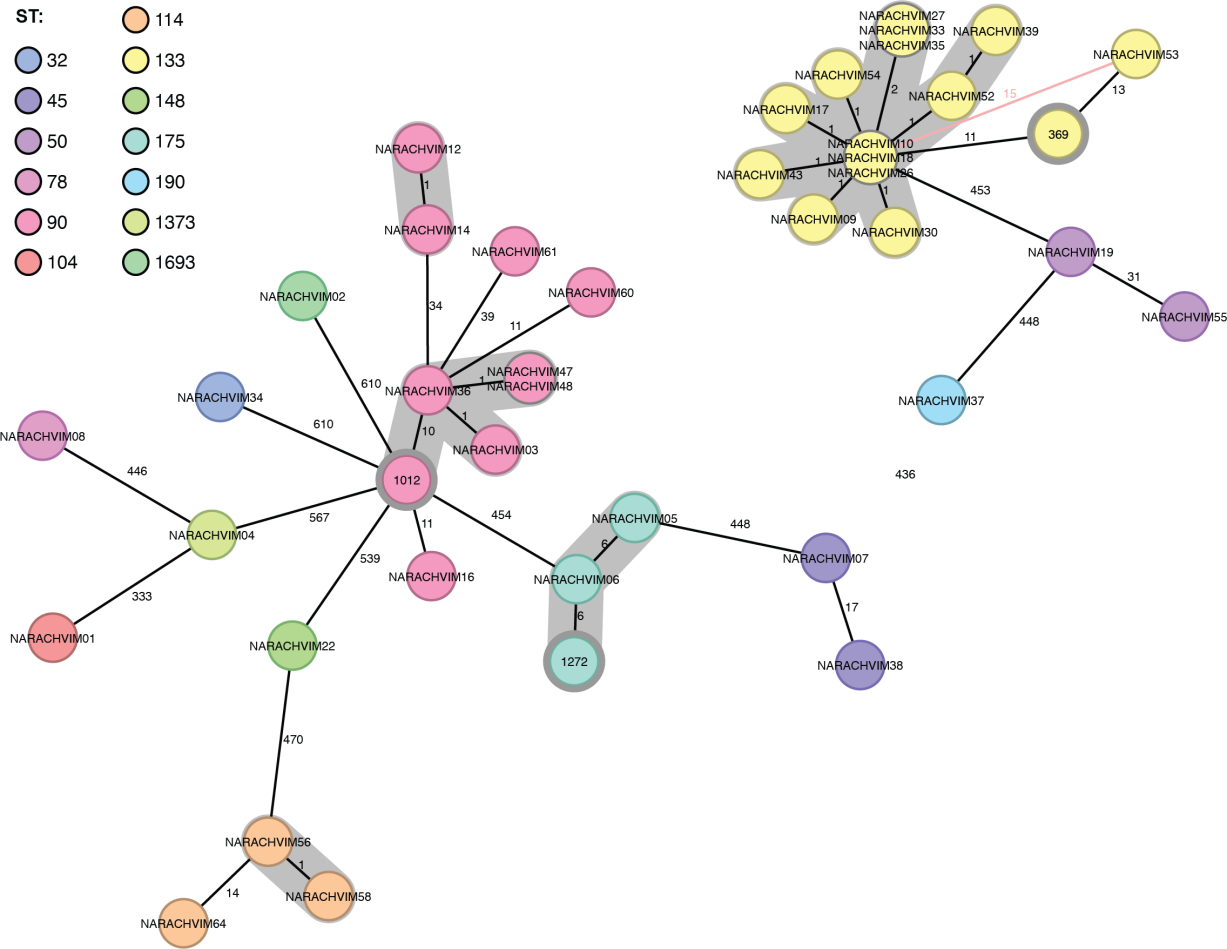
Minimum spanning tree of *Enterobacter* spp. based on 638-locus cgMLST. Isolates are colored by ST, and allele distances are shown on the edges. Clusters are shown with grey shadowing and were defined at the 10-allele threshold. Study isolates are shown, as well as three closely related isolates from databases (gray circles).

The higher-resolution cgMLST typing showed several clusters, the largest being of 13 genomes in ST133 differing by a maximum of two alleles, which represents up to 90 SNPs. The genome of NARACHVIM53 is 15 alleles distant, which represents a distance of over 1,000 SNPs across the genome. Two clusters were identified within ST90 (comprising four and two isolates, respectively, separated by 24 alleles, with another genome 12 alleles distant) and one cluster in ST175 (two isolates) (Fig. 2). A further 12 genomes belong to 10 STs and are unrelated to each other, including *E. kobei* ST32 and *E. ludwigii* ST1693.

Where replicate samples from patients were sequenced, they all clustered with a maximum of one allele difference in the cgMLST analysis (data not shown).

Searches for genomes related to the clusters within the PubMLST database (pubmlst.org) identified an ST133 isolate from Spain (369; date unknown), 11 alleles from the cluster; an ST90 isolate from Australia (1012; 2018), 10 alleles from the larger ST90 cluster; and one ST175 genome from China (1272; 2015), six alleles from the cluster.

### Antimicrobial resistance determinants

In addition to the *bla*_VIM-1_ gene, the isolates carried a range of further antimicrobial resistance determinants (Table S2). Duplicate patient samples showed largely the same profiles, but with some loss and gain of determinants (Table S2). Genomic virulence factors are presented in the Supplementary Information.

### Plasmids carrying the *bla*_VIM-1_ gene

**Fig 3:**
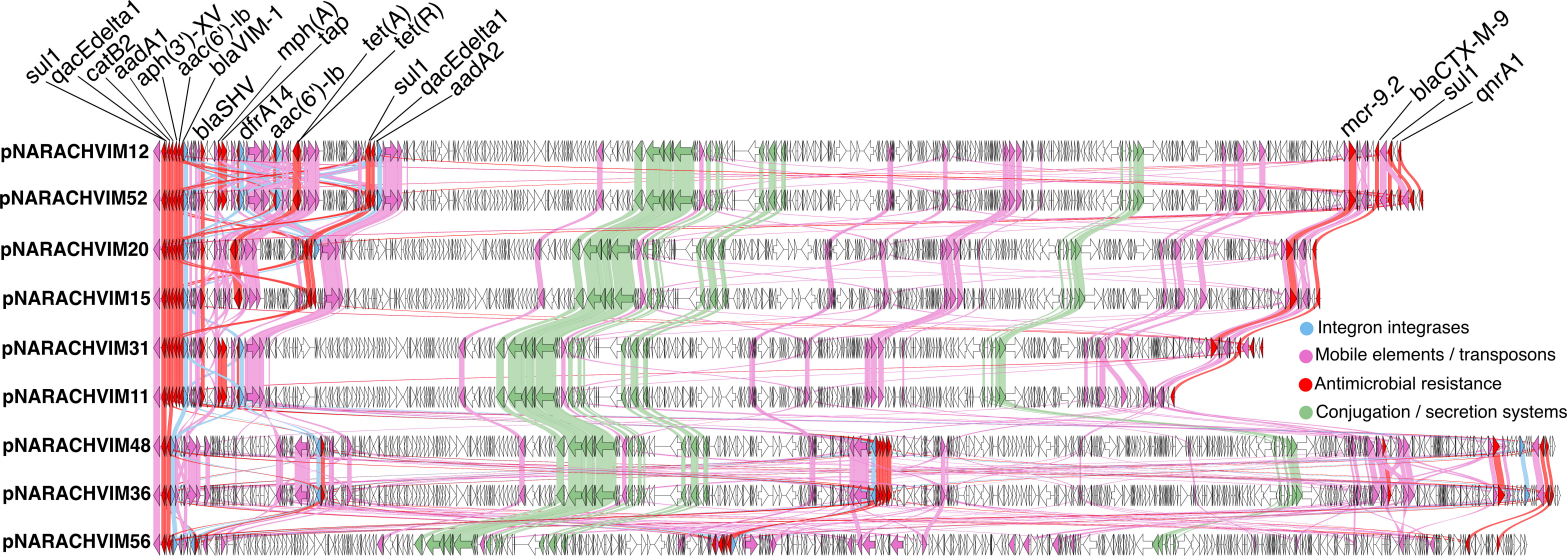
Comparison of IncHI2 plasmids showing insertions, deletions, and rearrangements. Annotation of pNARACHVIM12 shows ARGs in red, genes related to conjugation/transfer in green, integron integrases in dark blue, and transposases/phage genes in pale pink. pNARACHVIM12 is from ST90; pNARACHVIM52, pNARACHVIM20, pNARACHVIM15, and pNARACHVIM31 are from ST133; pNARACHVIM11 is from ST175; pNARACHVIM48 is from ST45; pNARACHVIM36 is from ST90; and pNARACHVIM56 is from ST114. Gene names of ARGs are shown. The figure was generated using Clinker v0.0.31 (29) (github.com/gamcil/clinker) and Genbank annotation files adapted from IMMense output.

Hybrid assemblies of 12 selected isolates showed that in the majority of isolates, the *bla*_VIM-1_ gene was located on an IncHI2 plasmid ranging in size from 249 kb (pNARACHVIM11, within ST175) to 343 kb (pNARACHVIM36, within ST90). In all these plasmids, the *bla*_VIM-1_ gene was found as a gene cassette located at the first position of a class 1 integron, which also carried *cat*B2, *sul*1, *qnr*A1, and aminoglycoside transferases (Fig. 3). Most of these plasmids also carried the *mcr-9* gene, associated with colistin resistance, which was not located adjacent to the *qseC* and *qseB* genes, possibly indicating a lack of expression (28). Several of the plasmids carry the *tet*(A) gene, encoding resistance to tetracycline, which is associated with an IS*6* family IS element (Fig. 3). Toxin-antitoxin systems can also be found on the plasmids, including those with homology to *hok-sok* and *relE-relB*. Among the nine complete hybrid-assembled IncHI2 plasmids, diversity in the plasmid gene content was apparent (Fig. 3).

Three *E. hormaechei* isolates—NARACHVIM07, NARACHVIM08, and NARACHVIM61—belonging to ST45, ST206, and ST90, respectively, did not carry IncHI2 plasmids and were also investigated using hybrid genome methods. In these isolates, *bla*_VIM-1_ was located on distinct circular plasmids: an IncN plasmid of approximately 68 kb in NARACHVIM07, an IncA plasmid of approximately 176 kb in NARACHVIM08, and an IncM1 plasmid of approximately 82 kb in NARACHVIM61. Comparisons of these against their closest related published plasmids show a potential gain of the *bla*_VIM-1_-containing region in pNARACHVIM61, but existing arrays of AMR genes in the other plasmid types (Fig. S1).

### IncHI2 plasmid conjugation

To demonstrate the self-conjugation of the IncHI2 plasmids, two isolates NARACHVIM20 and NARACHVIM36 were conjugated with susceptible *E. coli* J53 hosts. A very low-frequency plasmid conjugation of 3.5 × 10^−10^ was observed, and transconjugants were confirmed by antimicrobial sensitivity testing (Fig. S2) and PCR amplification of *bla*_VIM-1_. Some differences were observed in the resistance profiles conferred by the two plasmids in terms of resistance to tetracycline, chloramphenicol, and trimethoprim-sulfamethoxazole.

### Comparisons of IncHI2 plasmids

A first step in IncHI2 plasmid comparisons was to use a network-based method to group the hybrid-assembled plasmids (Fig. S3). Three clusters were apparent, reflecting groups of closely related plasmids. Within the largest cluster, which consists of plasmids from isolates belonging to ST90, ST133, and ST175, high similarity (containment 0.00–0.02) and minimal rearrangement were observed (DCJ-Indel ≤ 6), whereas between-cluster edges show higher measures of diversity (containment 0.13–0.22) and structural variation (DCJ-Indel 33-49). Pling analysis of the IncN, IncA, and IncM1 plasmids also showed that these do not cluster with each other or with the IncHI2 plasmids (data not shown).

**Fig 4:**
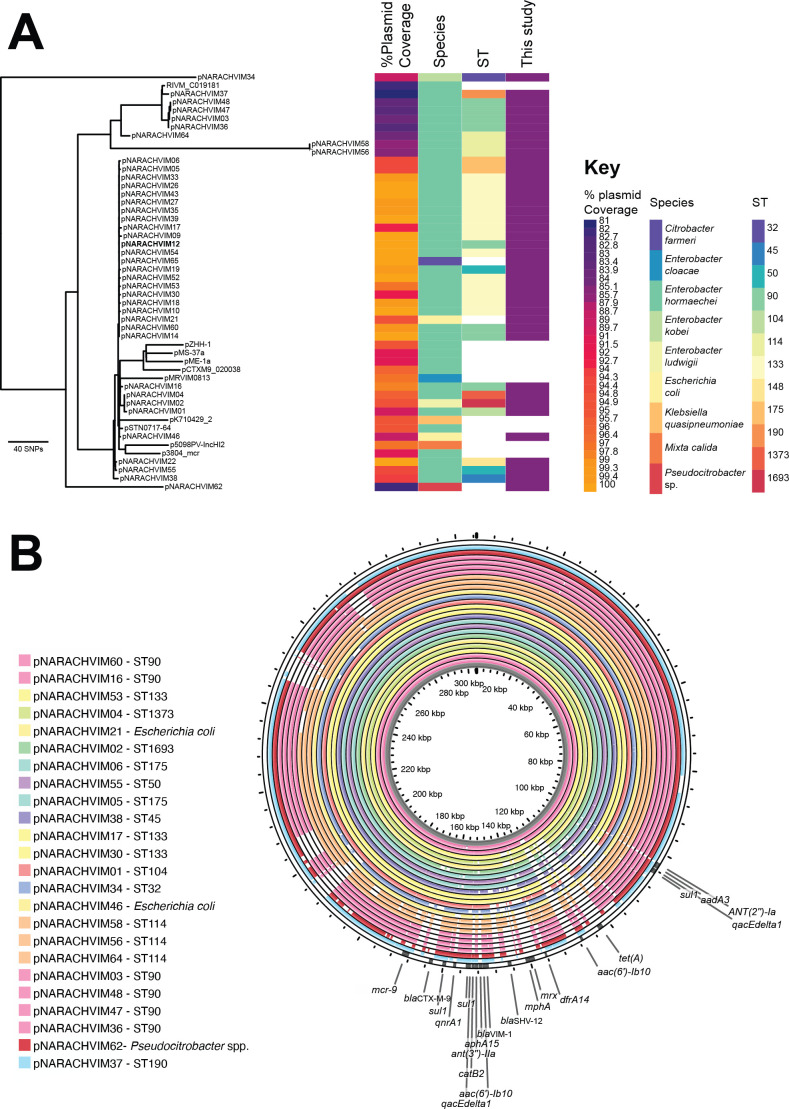
Variation of *bla*_VIM-1_-carrying IncHI2 plasmids. (**A**) Phylogenetic tree of highly related *bla*_VIM-1_-carrying plasmids. Sequence data from the study were compared also to reference plasmids from the literature. Reads from all isolates were mapped against the hybrid assembly of the plasmid from isolate pNARACHVIM12 (bold), and those with over 70% coverage are displayed. The % coverage of the plasmid is given, as are the isolate species and *Enterobacter* MLST. Figure generated in Phandango (30). (**B**) Comparison of plasmid content across *Enterobacter* STs. Plasmids from the study are ordered by % identity and colored by sequence type. Variation in plasmid content between isolates can be seen. Figure generated in Proksee (31) based on the reference plasmid pNARACHVIM12 and Illumina assemblies. ARGs for this figure were identified using CARD (32).

A plasmid phylogeny was generated to investigate nucleotide identities between the highly similar IncHI2 plasmids (Fig. 4A). The reference plasmid selected was pNARACHVIM12, an early (October 2022) and full-length 304 kb representative of the largest cluster. Coverage of the reference plasmid based on mapped Illumina data were also calculated as a metric of gene loss. Of the 39 isolates, 36 mapped to the reference with >80% coverage and >99.9% nucleotide identity (max 300 SNPs across the shared plasmid regions), and within these, there are also clades of plasmids with very few SNPs. What varies, in addition to nucleotide substitutions within these clades, is gene content, with plasmid coverage in the larger cluster as low as 88.7% (pNARACHVIM46 compared to the reference pNARACHVIM12) and 83% in the smaller cluster of five closely related plasmids (pNARACHVIM37 compared to the reference pNARACHVIM36; data not shown).

The transfer of the IncHI2 plasmids between *Enterobacter spp*. STs, *Enterobacter* spp., and even to species other than *Enterobacter* is suggested from the phylogeny. Highly identical IncHI2 plasmids from the larger cluster are found in *E. hormaechei* ST45, ST50, ST90, ST104, ST133, ST148, and ST1373, and *E. ludwigii* ST1693, as well as in contemporaneous isolates of *Citrobacter farmeri* (NARACHVIM65) and *E. coli* (NARACHVIM21 and NARACHVIM46) (Fig. 4A). Plasmids in the smaller cluster were found in isolates of *E. hormaechei* ST90 and ST190, and a slightly more distantly related plasmid was also found in a possibly new species of *Pseudocitrobacter* spp. (NARACHVIM62).

Previously described IncHI2 plasmids are also closely related (Fig. 4A), in particular pSTN0717-64 (DRR199787) from *E. hormaechei* isolated in Japan in 2018 and pRIVM_C019181 (CP071016) from *E. hormaechei* isolated in the Netherlands in 2018. Regions of the plasmid lost over the course of the study period include *qnrA1*, *mph*(A), *dfrA14*, *tet*(A), *bla*_CTX-M-9_, and *mcr-9* (Fig. 4B).

Plasmid gene variation also occurred within-host, with gene loss being observed in repeat patient samples (Fig. S4). Regions absent in the second isolate but present in the first correlate with loss of ARGs (Table S2). These changes can also occur over very short timespans (20 days between isolates NARACHVIM06 and NARACHVIM11; 68 days between isolates NARACHVIM09 and NARACHVIM15). In other cases, the plasmid content remained stable over up to 78 days (NARACHVIM22 to NARACHVIM29).

## DISCUSSION

We describe a multiclonal dissemination of *bla*_VIM-1_-carrying Enterobacterales in Switzerland, partly driven by multi-ARG-carrying IncHI2 plasmid transmission between STs and species. No further cases were detected after October 2024, suggesting that this spread has subsided, perhaps reflecting increased awareness and hospital hygiene measures. The precise sources and transmission events remain unclear, both with respect to international strains and plasmids and within Switzerland.

Closely related plasmids were also identified in three further species, indicating possible interspecies as well as intraspecies plasmid transmission. In most isolates, IncHI2 plasmids were found to carry the *bla*_VIM-1_ gene and a host of additional ARGs, including *mcr-9*. Two variants of an IncHI2 plasmid were identified in the vast majority of the ECC isolates, with long-read sequencing being key in this aspect of the investigation. Additionally, three isolates (NARACHVIM07, NARACHVIM08, and NARACHVIM61) carried *bla*_VIM-1_ on distinct IncN, IncA, and IncM1 plasmids unrelated to the predominant IncHI2 lineage. This highlights the background diversity of *bla*_VIM-1_ genetic contexts in co-circulating isolates. Despite differences in plasmid backbone structure, the structure of the *bla*_VIM-1_-containing resistance cassette was largely conserved across plasmid types, suggesting either a common cassette arrangement or a possible mobilization of this cassette between otherwise unrelated plasmid backbones.

The findings of the predominant IncHI2 lineage can be placed in the context of previous reports of IncHI2 plasmids carrying carbapenemase genes and *mcr-9* in Europe, including in *Enterobacter cloacae* complex isolates from the Netherlands (33), in *Enterobacter hormaechei* from non-European settings (16), and in other Enterobacterales such as *Klebsiella pneumoniae* (34). In Switzerland, IncHI2 plasmids carrying *bla*_VIM-1_ and *mcr-9* have been described in other Enterobacterales species, notably *Klebsiella grimontii* (35), but not in *Enterobacter* spp. to date. Our findings therefore provide a detailed characterization of IncHI2-mediated *bla*_VIM-1_ dissemination in *Enterobacter* spp. within a national Swiss surveillance context.

IncHI2 conjugative plasmids are strongly linked to *mcr-9* and beta-lactamase genes. Of the previously published related plasmids, all carry a battery of ARGs including *mcr-9* genes, along with *bla*_NDM-1_ in pK710429 (China) (34), *bla*_SHV-12_ in 5089PV (Italy) (36), and *bla*_CTX-M-9_ in pSTN0717-64 (Japan, accession AP022511). The *bla*_VIM-1_ gene is found along with *mcr*-9 on pRIVM_C019181 (the Netherlands) (33). The speed at which such plasmids can undergo nucleotide substitutions and rearrangements is not known. The observation of plasmids with very few SNPs and only minor rearrangements over the study period suggests recent common ancestry of these plasmid variants within the investigated data set. Loss of ARGs over time, both across isolates collected during the study period and within individual hosts, suggests that not all resistance determinants were under sustained selective pressure and reinforces the impression of plasmid plasticity, even over short timescales. Indeed, given this plasmid plasticity, it is possible that the same plasmid backbone might also have lost the *bla*_VIM-1_ gene in the absence of selective pressure, but any such isolates would not have been included in this study as the presence of the *bla*_VIM-1_ gene was a prerequisite for inclusion.

This study shows the relevance of plasmid analysis for understanding transmission dynamics and underscores the importance of genomic surveillance in the context of carbapenemase-carrying and multidrug-resistant isolates. It raises awareness of scenarios in which plasmid-mediated dissemination may not be apparent from chromosomal typing alone. For detailed plasmid analysis, both short- and long-read sequencing is required and can thus offer extremely high levels of resolution, at chromosome, plasmid, and ARG scales.

## Data Availability

All read data were submitted to the European Nucleotide Archive (ENA) under project number PRJEB98563 (Table S2).

## SUPPLEMENTAL MATERIAL

Supplemental figures aac.01827-25-s0001.docx Fig. S1 to S5.

Table S1 aac.01827-25-s0002.xlsx ONT sequencing QC per isolate.

Table S2 aac.01827-25-s0003.xlsx Features of isolates and submitted sequencing data.

Supplementary Information aac.01827-25-s0004.docx Virulence factors.

ASM does not own the copyrights to Supplemental Material that may be linked to, or accessed through, an article. The authors have granted ASM a non-exclusive, world-wide license to publish the Supplemental Material files. Please contact the corresponding author directly for reuse.
