# Supplementary material for: Investigation of VIM-1-producing Enterobacter spp. across Switzerland: clonal dissemination and plasmid transmission
Source: Antimicrob Agents Chemother. 2026 May 5;70(6):e01827-25. doi: 10.1128/aac.01827-25 (PMC13231877; doi:10.1128/aac.01827-25)
Supplement: Supplemental figures — Fig. S1 to S5. [file aac.01827-25-s0001.docx]

**Supplementary Figures**

**
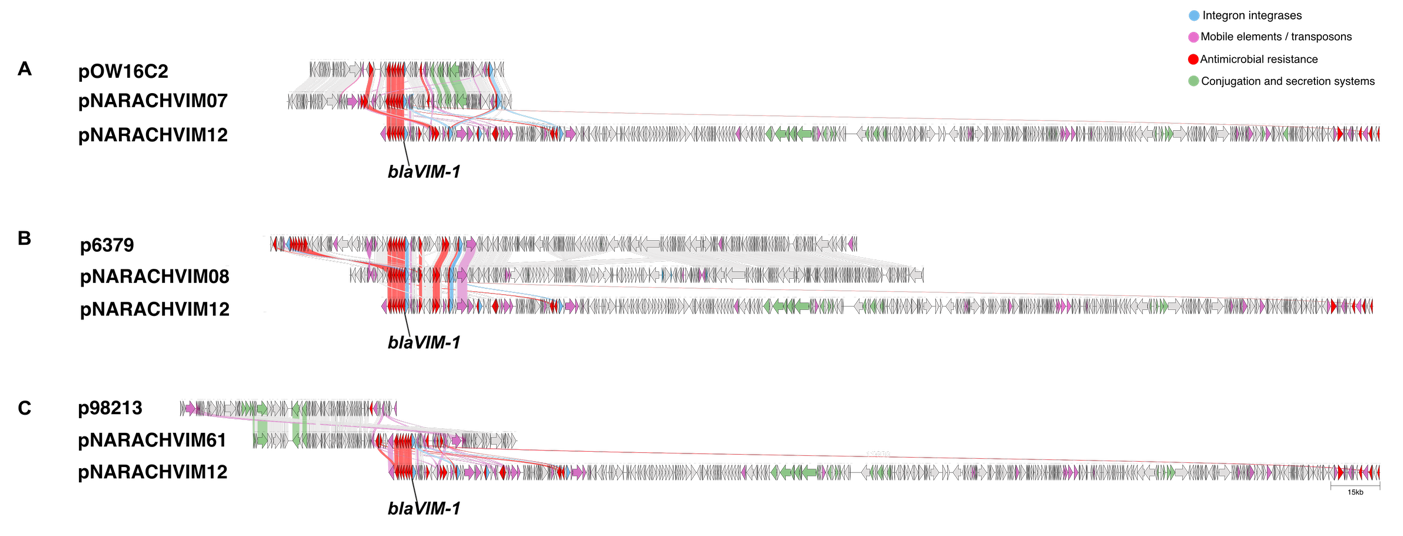
**

**Figure S1. Comparison of non-IncHI2 plasmids against reference plasmid pNARACHVIM12 and closest related plasmids from the databases.**

**A.** The IncM1 pNARACHVIM07 showed highest similarity to plasmid pOW16C2 (KF977034: 88% query cover, 100% identity) identified by NCBI BLAST and differed from the IncHI2 outbreak plasmid backbone.
**B.** Similarly, the IncA plasmid from NARACHVIM08 was most closely related to plasmid p6379 (OQ282880: 78% query cover, 100% identity), again displaying a backbone distinct from the IncHI2 outbreak plasmid.
**C.** The IncN plasmid from NARACHVIM61 likewise showed highest similarity to plasmid p98213 (CP173520: 72% query cover, 99.99% identity) rather than to the IncHI2 outbreak plasmid. Despite these differences in plasmid backbone, the *bla*_VIM-1_-containing region was highly conserved across plasmid types. Figure was generated using Clinker v0.0.31 [29] ([github.com/gamcil/clinker](http://github.com/gamcil/clinker)) and Genbank annotation files adapted from IMMense output.

**
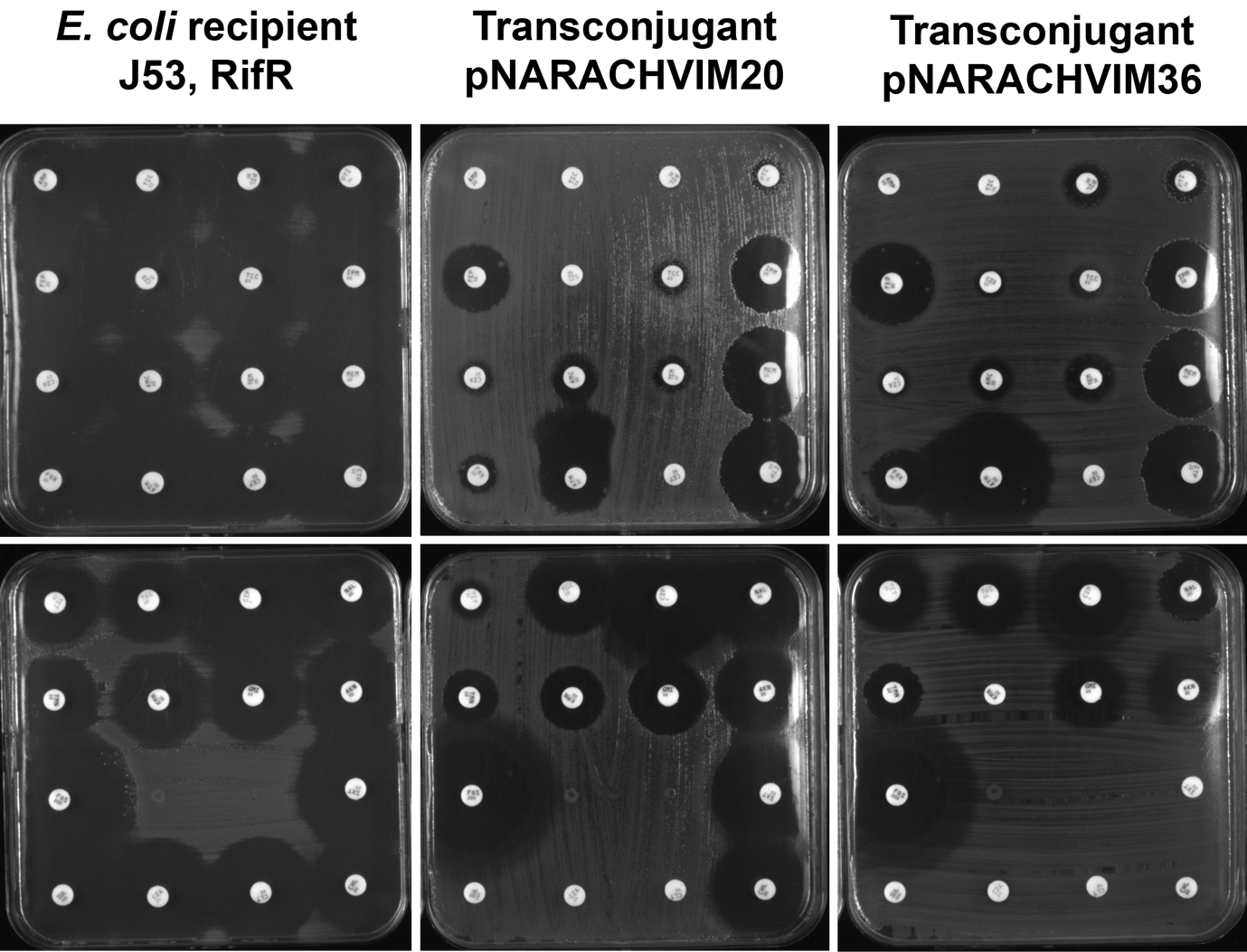
**

**Figure S2. Plasmid conjugation and antimicrobial resistance transfer.** AST profiles for trans-conjugants of *E. coli* J53 recipient and donors NARACHVIM20 and NARACHVIM36, highlighting differences in resistance conferred by the two IncHI2 plasmids. Top plates antibiotic discs are (top left to top right, then second row left to right etc): AMP, ampicillin; TIC, ticarcillin; PIL, pipercillin; PTZ, pipercillin-tazobactam; FEP, cefepime; CZD, ceftazidime; TCC, ticarcillin-clavulanic acid; IPM, imipenem; CZA, ceftazidime-avibactam; AMC, amoxicillin-clavulanic acid; TEM, temocillin; MEM, meropenem; FOS, fosfomycin; ATM, aztreonam; CEF, cefalothin; ETP, ertapenem. Bottom plates antibiotic discs are (top left to top right, then second row left to right etc): TET, tetracycline; TGC, tigecycline; CIP, ciprofloxacin; NAL, nalidixic acid; TMN, tobramycin; KMN, kanamycin; GMI, gentamicin; AKN, amikacin; FOS, fosfomycin; SXT, trimethoprim-sulphamethoxazole; SUL, sulphamethoxazole; CZA, ceftazidime-avibactam; CLT, ceftolozane-tazobactam; CHL, chloramphenicol. Phenotypic differences between the two transconjugant backgrounds were most evident for tetracycline, kanamycin, chloramphenicol and trimethoprim-sulfamethoxazole. Phenotypic differences generally align with gene content (e.g. *tet(*A) in pNARACHVIM20 explaining TET-R; *dfrA*19 in pNARACHVIM36 explaining SXT-R) NB for chloramphenicol, although pNARACHVIM20 carries *cat*B2 both the parent strain NARACHVIM20 and the transconjugant remain susceptible, suggesting a non-functional gene.

*
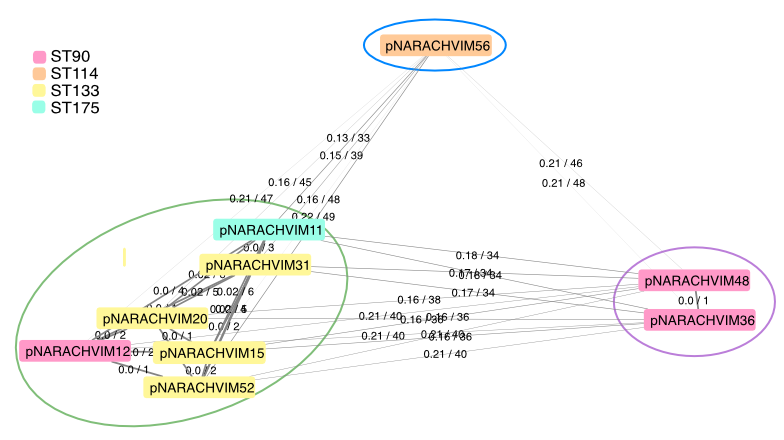
*

**Figure S3. Graph of plasmid network.** Figure was generated with pling v2.0.1. Nodes represent plasmids and are coloured by genome ST and edges labelled with the values “containment / DCJ-Indel” to facilitate interpretation. The pairwise containment distances range from 0.00 to 0.22. DCJ-Indel distances span 1 to 49 rearrangement or indel events, indicating variable structural divergence. The nodes segregate into three visually distinct clusters circled in green, purple, and blue.


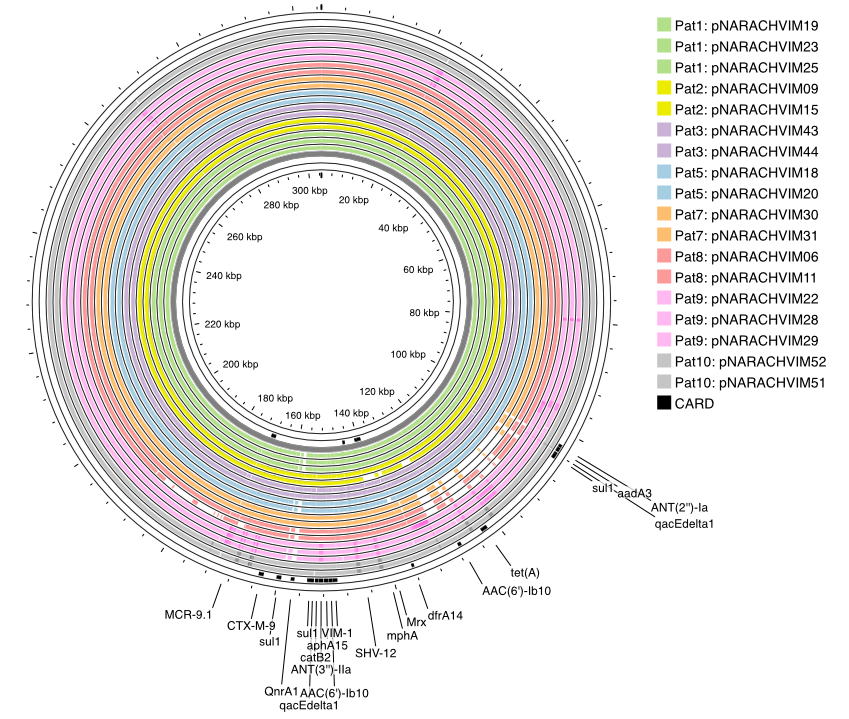


**Figure S4. Comparison of plasmid content from repeat patient isolates.** All plasmids in isolates from the same patients showed >99% nucleotide identity. Plasmids are coloured by the patient, and earlier isolates are shown further towards the centre of the circle. Some variation in plasmid content between isolates can be seen in Patients 2, 5 and 8. Figure generated in Proksee [27] based on the reference plasmid pNARACHVIM12 and Illumina assemblies. ARGs for this figure were identified using CARD [28].


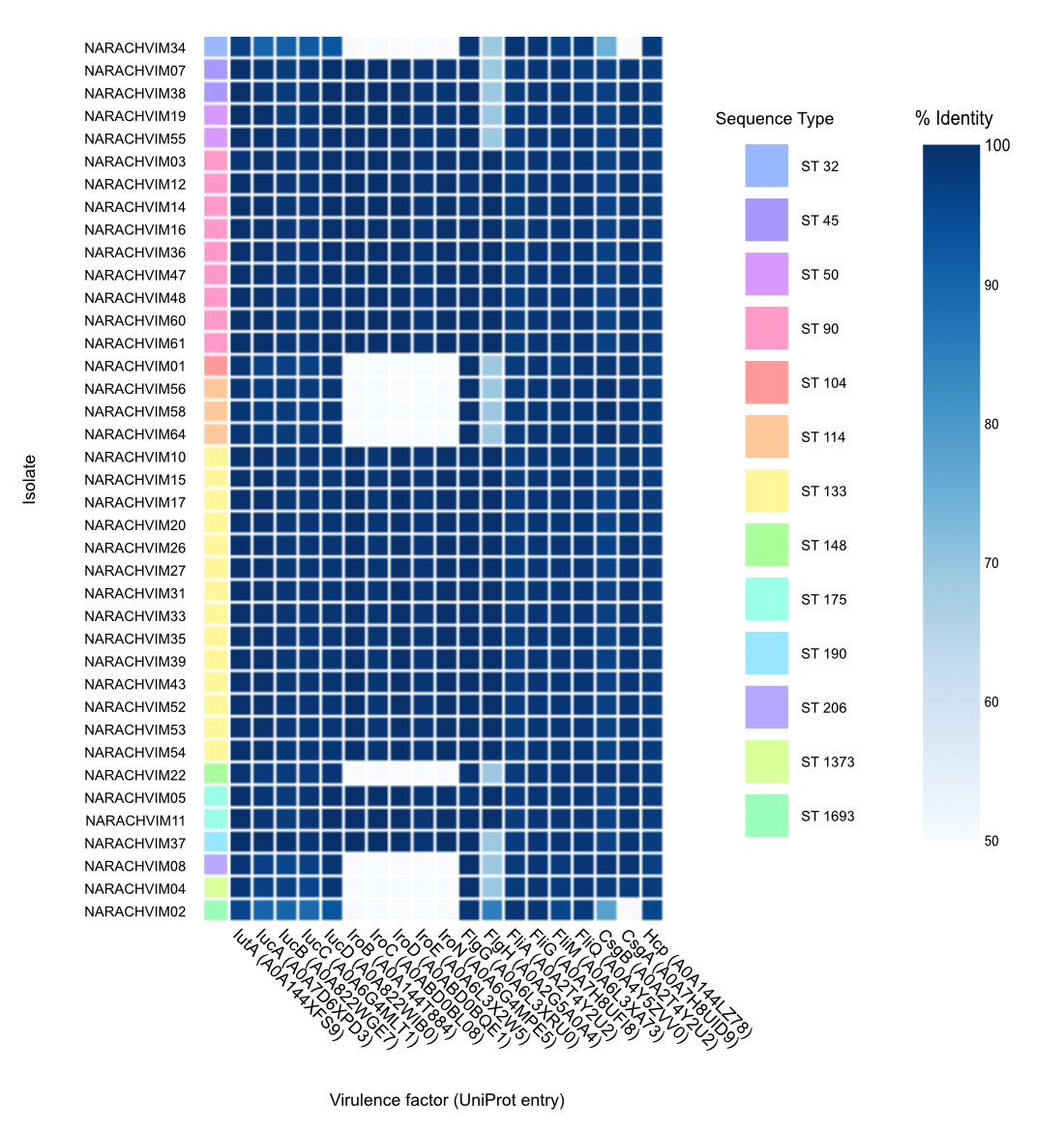


#### **Figure S5. Heatmap showing the presence of selected virulence factors within analysed isolates.** Percent amino-acid identity of the top TBLASTN hit in each genome against reference virulence-factor proteins. Columns: virulence factors (UniProt entry IDs in parentheses). Rows: isolates, ordered by sequence type (ST). Identity values range from 50-100%. White cells mark alignments below the thresholds (coverage ≥ 0.9, identity ≥ 50%).
