# Supplementary material for: Investigation of VIM-1-producing Enterobacter spp. across Switzerland: clonal dissemination and plasmid transmission
Source: Antimicrob Agents Chemother. 2026 May 5;70(6):e01827-25. doi: 10.1128/aac.01827-25 (PMC13231877; doi:10.1128/aac.01827-25)
Supplement: Supplementary Information — Virulence factors. [file aac.01827-25-s0004.docx]

**Supplementary Information**

*Virulence factors*

Reference protein sequences for six relevant virulence-associated operons (*iutAiucABCD, iroBCDEN, flgGH, fliAGMQ, csgBA,* and *hcp*) [1] were retrieved from the UniProt database. TBLASTN v2.13.0 [2] was used to search genome assemblies against this database. Results were filtered based on coverage (0.9 to 1) to eliminate non-specific matches. Hits with sequence identity below 0.5 were excluded.

The presence of six key virulence-associated operons was investigated across the 39 clinical *Enterobacter* isolates (Figure S5). The genes include *iutA-iucABCD* (aerobactin siderophore system), *iroBCDEN* (salmochelin siderophore system), *flgGH* and *fliAGMQ* (flagella assembly), *csgBA* (curli fimbriae), and *hcp* (hemolysing-coregulated protein).

All the virulence factors were found in all isolates of ST133, ST90 and ST175. An alternative *flgH* locus is apparent in isolates of ST45, ST50 and ST190. The *iroBCDEN* operon is absent from isolates of ST104, ST114, ST148 and ST1373. This operon and *csgA* are not present in isolates of ST32 and ST1693.

**References**

1. Huang Y, Wu Y, Cai C, Zhang R, Chen G, Dong N. Phenotypic and Genomic Characterization of ST133 Siderophore-Encoding Extensively Drug-Resistant Enterobacter hormaechei. Antimicrob Agents Chemother. 2023 Apr 18;67(4):e0173722.

2. Camacho C, Coulouris G, Avagyan V, Ma N, Papadopoulos J, Bealer K, et al. BLAST+: architecture and applications. BMC Bioinformatics. 2009 Dec;10(1):421.
